# Collagen Peptides Derived from *Sipunculus nudus* Accelerate Wound Healing

**DOI:** 10.3390/molecules26051385

**Published:** 2021-03-04

**Authors:** Haisheng Lin, Zhihong Zheng, Jianjun Yuan, Chaohua Zhang, Wenhong Cao, Xiaoming Qin

**Affiliations:** 1 Key Laboratory of Inshore Resources Biotechnology (Quanzhou Normal University), Fujian Province University, Quanzhou 362000, China; haishenglin@163.com; 2 College of Food Science and Technology, Guangdong Ocean University, Zhanjiang 524088, China; zhzheng617@163.com (Z.Z.); cchunlin@163.com (W.C.); qinxm@gdou.edu.cn (X.Q.); 3 Guangdong Provincial Key Laboratory of Aquatic Product Processing and Safety, Zhanjiang 524088, China; 4 Guangdong Province Engineering Laboratory for Marine Biological Products, Zhanjiang 524088, China; 5 Guangdong Provincial Engineering Technology Research Center of Marine Food, Zhanjiang 524088, China; 6 Key Laboratory of Advanced Processing of Aquatic Product of Guangdong Higher Education Institu-tion, Zhanjiang 524088, China; 7 Collaborative Innovation Center of Seafood Deep Processing, Dalian Polytechnic University, Dalian 116034, China

**Keywords:** *Sipunculus nudus*, collagen peptides, wound healing, inflammation, scar inhibition, collagen, transforming growth factor

## Abstract

Marine collagen peptides have high potential in promoting skin wound healing. This study aimed to investigate wound healing activity of collagen peptides derived from *Sipunculus nudus* (SNCP). The effects of SNCP on promoting healing were studied through a whole cortex wound model in mice. Results showed that SNCP consisted of peptides with a molecular weight less than 5 kDa accounted for 81.95%, rich in Gly and Arg. SNCP possessed outstanding capacity to induce human umbilical vein endothelial cells (HUVEC), human immortalized keratinocytes (HaCaT) and human skin fibroblasts (HSF) cells proliferation and migration in vitro. In vivo, SNCP could markedly improve the healing rate and shorten the scab removal time, possessing a scar-free healing effect. Compared with the negative control group, the expression level of tumor necrosis factor-α, interleukin-1β and transforming growth factor-β1 (TGF-β1) in the SNCP group was significantly down-regulated at 7 days post-wounding (*p* < 0.01). Moreover, the mRNA level of mothers against decapentaplegic homolog 7 (Smad7) in SNCP group was up-regulated (*p* < 0.01); in contrast, type II TGF-β receptors, collagen I and α-smooth muscle actin were significantly down-regulated at 28 days (*p* < 0.01). These results indicate that SNCP possessed excellent activity of accelerating wound healing and inhibiting scar formation, and its mechanism was closely related to reducing inflammation, improving collagen deposition and recombination and blockade of the TGF-β/Smads signal pathway. Therefore, SNCP may have promising clinical applications in skin wound repair and scar inhibition.

## 1. Introduction

Skin wounds are mainly caused by external forces, such as accidents, burns, traumas and genetic diseases, resulting in the destruction of skin tissue and cell arrangement, rupture of blood vessels and loss of blood [1]. Wounds are easily infected after suffering injury and in the absence of good care, these could easily develop into chronic wounds (such as diabetic ulcers). This can cause severe physical dysfunction and mental damage among patients. In total, approximately 2% of people in the United States suffer from chronic wounds, and the annual cost of wound care is between 28.1 billion and 96.8 billion US dollars [2]. 

Skin wound healing is a relatively complex biological repair process, including four continuous and overlapping phases: blood clotting, inflammation, proliferation and tissue remodeling [3,4]. Hemostasis and recovery of the vascular system, reduced inflammation, promotion of fibroblast differentiation and connective tissue formation are of great significance in wound healing [3]. A number of drugs and health care products that promote wound healing are mainly designed according to the four stages on which intervene, such as hemostatic drugs, antimicrobial drugs, anti-inflammatory drugs or agents that promote collagen synthesis. Recent research demonstrated that the decrease of inflammation and highly orchestrated remodeling could improve the performance of new regenerative skin, and even achieve scarless healing [5,6], and many Chinese herbal medicine and marine bioactivity substances, such as collagen (peptides), have been shown to accelerate skin wound healing and prevent abnormal scar formation [7,8,9].

Marine collagen has been separated from numerous marine resources, some of which has the effect of promoting wound healing, such as *Sipunculid* collagen and fish collagen [8,10,11]. Recent reports revealed that biomaterials based on collagen, such as collagen powder, collagen gels, collagen sponges and collagen dressings, possess numerous biological functions of wound healing [12,13,14]. Compared with collagen, marine collagen peptides with lower molecular weight are easy to absorb, and possess strong affinity to water as well as interesting biological functions, such as antioxidant, inflammatory, antifreeze, angiotension-I converting enzyme inhibition (ACE-I), antimicrobial, anti-aging and wound healing activities [15,16]. Further studies of collagen peptides revealed that certain marine collagen peptides have high potential in promoting skin wound healing, such as sea cucumber collagen, salmon (*Oncorhynchus keta*), salmo salar and *Tilapia nilotica* skin collagen peptides [7,15,17,18], but there is no related report on the promotion of wound healing by *Sipunculus nudus* collagen peptides.

*Sipunculus nudus* is a widespread economic benthic organism in the coastal areas of southern China. It is rich in nutrients and has a high content of arginine, which possess the function of promoting wound healing [6,19]. Various researches suggested that *Sipunculus nudus* had anti-oxidation, anti-radiation, immune regulation, anti-bacterial, anti-inflammation and peripheral analgesia effects [20,21,22,23,24,25]. Our previous studies have demonstrated that the *Sipunculus nudus* extract possessed obvious activity of accelerating wound healing and inhibiting scar formation, and its mechanism was related to its anti-inflammation function [6]. It was reported that the coelomic wall of *Sipuncula* animal is rich in collagen, but with poor water solubility [11,26]. Enzymatic hydrolysis could be an effective method for preparation of collagen peptides. By controlling hydrolyzing condition, collagen hydrolysate with small molecular has advantages of excellent water solubility and good transdermal absorption, and it might be a suitable candidate for wound healing applications. However, the functions of *Sipunculus nudus* collagen peptides were still unclear. This study aimed to investigate the wound healing potential of collagen peptides prepared by enzymes. Here, we report the effects and related mechanism of *Sipunculus nudus* collagen peptides (SNCP) on promoting wound healing and preventing abnormal scar formation. Our findings show that SNCP could be used as a marine wound healing agent and provide a basis for its application in pharmaceutical fields. 

## 2. Results

### 2.1. The Extracted Collagen from Sipunculus nudus (SNC) is Type I 

**Figure 1:**
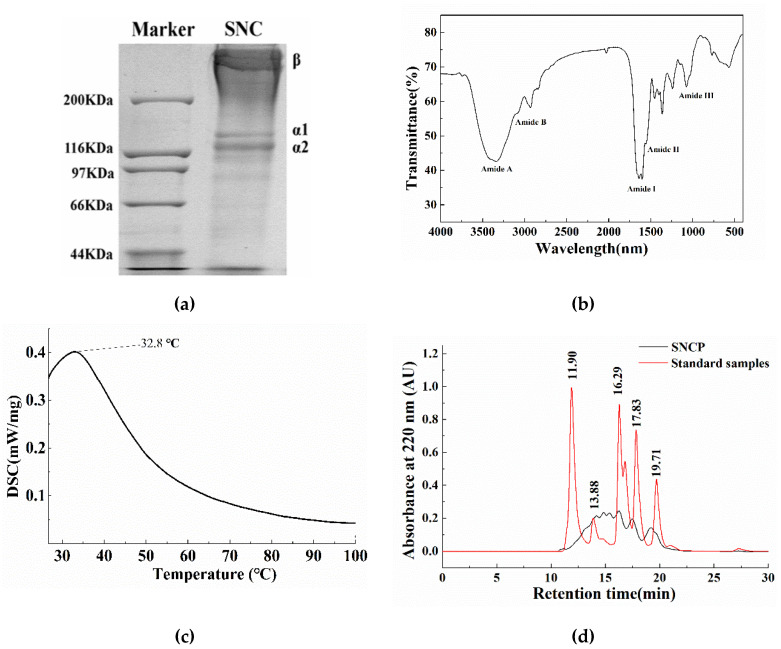
Properties of collagen (SNC) and peptides (SNCP) from *Sipunculus nudus*. (**a**) The electrophoretic patterns of SNC; (**b**) Fourier transform infrared spectrum of SNC; (**c**) Thermogram of SNC; (**d**) Chromatograms of mixed standard samples and SNCP, (from left to right) the standard samples were consisted of cyyochrome (12,500 Da), aprotinin (6500 Da), bacitacin (1450 Da), ethyl amino acid-ethyl amino acid-tyrosine-arginine (451 Da) and ethyl amino acid-ethyl amino acid-ethyl amino acid (189 Da) (in red).

The SDS-PAGE analysis showed that *Sipunculus nudus* collagen (SNC) comprises at least two different α chains and one β dimer (α1, α2, β), suggesting it is a typical type I collagen (Figure 1a). Therein, the molecular weight of α1 and α2 chains was approximately 130 kDa and 122 kDa, respectively. A previous study revealed that the acid-soluble collagen from the coelomic wall of *Sipunculida* was a type I collagen with α1 and α2 chains and a β dimer, in which the molecular weight of α1 and α2 was approximately 120 kDa and 110 kDa, respectively [26]. The different molecular weight of α chain in collagen might be due to the different class of *Sipuncula Sedgwick*. The patterns of the two α chains and one β dimer were similar to most collagens from fishes [27,28]. Type-I collagen could be applied in pharmaceutical, food and biomedical industries [29].

The Fourier transform infrared (FTIR) spectra of SNC exhibited five characteristic bands of the amide structure, namely A, B, I, II and III, as indicated in Figure 1b. The amide A band was found at 3340 cm^−1^, which indicates the stretching vibration of NH groups related to the hydrogen bonds. The amide B band found at 2931 cm^−1^ was the characteristic absorption of collagen different from other proteins, which was generally observed at 2930–2944 cm^−1^, causing by the asymmetric stretching vibration of -CH_2_. The strong absorption peak of amide I band (1600–1700 cm^−1^) was observed at 1638 cm^−1^ attributing to the C=O stretching vibration involved in the secondary structure of the peptide chain [30]. The amide II band and amide III band were mainly observed at around 1556 cm^−1^ and 1241 cm^−1^^,^ respectively, corresponding to the the N-H bending vibration and the in-phase C–N stretching vibration coupled with N–H bending vibration, which was responsible for collagen helical integrity [31,32]. The presence of the amide III band clearly demonstrated the existence of the triple helix structure in the SNC. Altogether, the FTIR spectra indicate a well-maintained secondary structure in SNC.

The differential scanning calorimeter (DSC) thermogram (Figure 1c) indicate that the denaturation temperature of SNC was 32.8 °C, which was similar to the acid-soluble collagen from carp fish scale waste (32.2 °C) [27], fresh carp fish scale (32.9 °C), giant grouper (31.7 °C) and big eye snapper (31 °C) [33,34]. It is reported that the thermal denaturation temperature of collagen was mainly related to the amino acid composition, protein conformation, water content, temperature of habitat and extraction method [32,35,36].

### 2.2. SNCP Consists of Peptides with Small Molecular Weight Less Than 5 kDa

The chromatogram of SNCP is shown in Figure 1d, respectively. With the retention time (t) as the horizontal axis and the lg Mw as the vertical axis, the regression equation lgMw = −0.2441t + 7.08 (R^2^ = 0.99) was obtained. The components of less than 1, 3, 5 and 10 kDa accounted for approximately 34.36%, 67.13%, 81.95% and 95.13%, respectively. Compared with SNC, SNCP with smaller molecular weight might possess better water solubility and facilitated absorption. 

### 2.3. SNCP is Rich in Gly and Arg

As shown in Table 1, Gly accounted for around 36% of the total hydrolyzed amino acid compositions, consistent with the Gly-X-Y repeating sequence of collagen [37,38]. Previous studies showed that Arg and Pro possessed the potential effect of promoting wound healing [19,39]. In this work, SNCP were also rich in Glx (Glu + Gln), Ala, Arg, Thr, Ser and Pro, which might contribute to the potential wound healing effect of SNCP. Moreover, the majority of the amino acid residues were hydrophilic, such as Glx (Glu + Gln), Arg, Asx (Asp + Asn) and Pro. This was consistent with the good water solubility of SNCP. The major free amino acids in SNCP were Arg (0.81 g/100 g), Leu (0.51 g/100 g), Tyr (0.42 g/100 g) and Pro (0.34 g/100 g); among them, Arg, which accounted for 19.36% of the total free amino acids, generally reflects the wound healing activity. 

### 2.4. SNCP Possesses an Outstanding Capacity to Induce HUVEC, HaCaT and HSF Cells Proliferation and Migration

**Figure 2:**
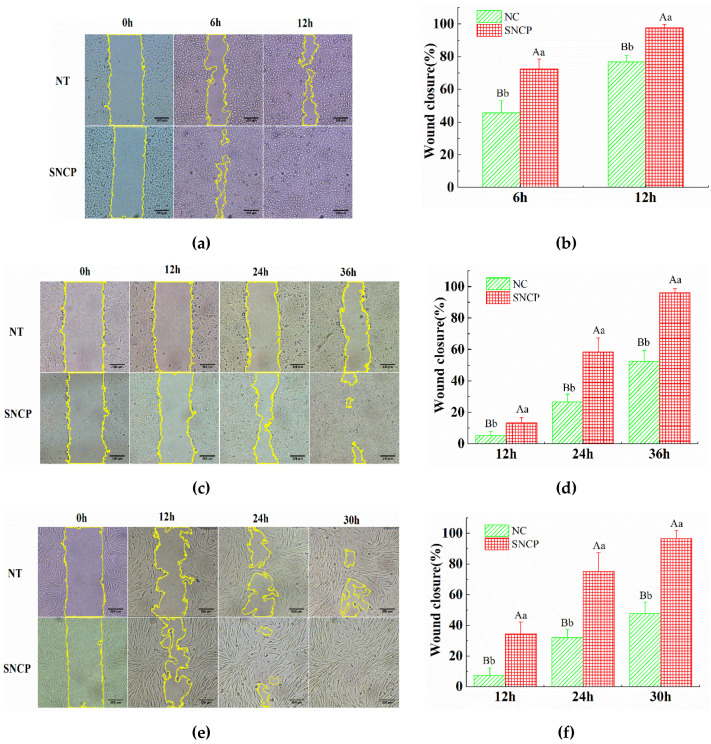
Effect of SNCP on wound closure assay in vitro. (**a**,**c**,**e**) Representative images of cell immigration and proliferation. (**b**,**d**,**f**) Wound closure percentages (n ≥ 6) in HUVEC endothelial cell (**a**,**b**), Hacat keratinocytes (**c**,**d**) and HSF fibroblasts (**e**,**f**). Scale bar = 200 μm. Values with different letters indicate significant differences in the different groups (capital letters: *p* < 0.01, small letters: *p* < 0.05). NC means negative control.

Endothelial cell, keratinocytes and fibroblasts are essential for effective angiogenesis, re-epithelialization and extracellular matrix (ECM) synthesis during the process of wound healing. In vitro scratch assay results showed that, compared with the negative control (NC) group, SNCP markedly promoted the wound closures by cell proliferation and migration in three cell strains, including human umbilical vein endothelial cells (HUVEC), human immortalized keratinocytes (HaCaT) and human skin fibroblasts (HSF) (Figure 2a,c,e). The wound closure percentages in HUVEC (97.54 ± 2.30%, in 12 h), HaCaT (96.13 ± 2.70%, in 36 h) and HSF cells (96.68 ± 5.08%, in 30 h), incubated with SNCP, were significantly higher than the ones of NC groups, respectively (*p* < 0.01) (Figure 2b,d,f). These results demonstrated that SNCP possessed outstanding capacity to induce HUVEC, HaCaT and HSF cells proliferation and migration. 

### 2.5. SNCP Accelerates the Wound Healing In Vivo 

**Figure 3:**
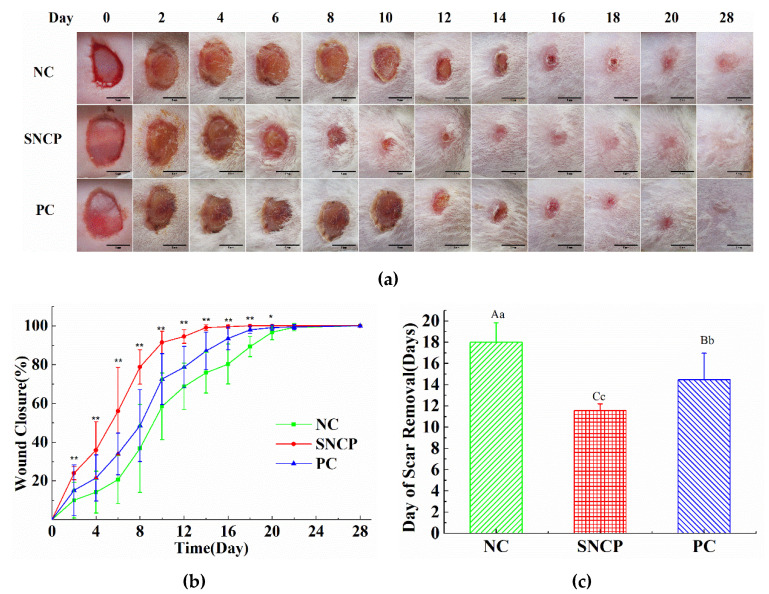
Skin wound healing over time in mice treated with SNCP. NC means negative control group in which wounds did not receive treatment, PC means positive control group, which was treated with Yunnanbaiyao powder (the same below). (**a**) Macroscopic observation of wounds in the excision wound model (scale bar = 5 mm). (**b**) Effect of SNCP on wound closure in mice, n ≥ 6; Compared with the NC group, statistical significance is found (SNCP, * *p* < 0.05, ** *p* < 0.01). (**c**) Time of scab removal, n ≥ 16; values with different letters indicate significant differences in the different groups (capital letters: *p* < 0.01, small letters: *p* < 0.05).

Figure 3 shows the effect of SNCP on wound healing in vivo. There was no significant difference observed in the wound in 2 days. The significant promoting effect of wound healing on SNCP was observed at 4 days after modeling. The mice groups treated with the SNCP had decreased wound area compared to the negative control (NC) group (Figure 3a). As shown in Figure 3b, a markedly higher wound closure rate was observed in SNCP group 2 days post-wounding, versus the NC group and positive control (PC) group, respectively. At 10 days post-wounding, wound closure rate of the SNCP group was (91.41 ± 5.77%), while for the NC group it was (58.48 ± 17.21%). The time of scab removal of each group is shown in Figure 3c. A markedly shorter time of scab removal was seen in the SNCP group (11.6 ± 0.6 d) versus in the PC group (14.5 ± 2.5 d) and in the NC group (18.0 ± 1.8 d). Importantly, SNCP group improved wound appearance. The surface of SNCP group in 28 days was smooth and flat without an obvious scar, whereas NC group developed noticeable large scars.

### 2.6. SNCP Reduces Excessive Inflammation Response

**Figure 4:**
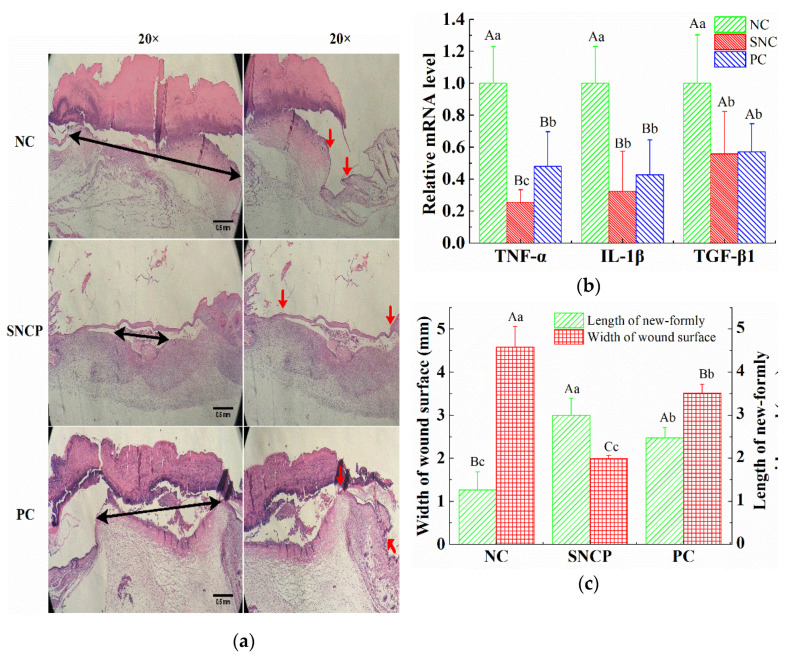
The effect of SNCP on wound surface and cytokines at 7 days post-wounding. (**a**) Histological analysis of the healing quality of wound epidermis. Black double arrows and red arrows represent the width of the wound surface and the newly-formed epidermis, respectively (scale bar = 0.5 mm). (**b**) Measurement of epidermal growth length and width of wound surface, n ≥ 4. (**c**) Expression of TNF-α, IL-1β and TGF-β1 in the wound of mice skin, n ≥ 4. Values with different letters indicate significant differences in the different groups (capital letters: *p* < 0.01, small letters: *p* < 0.05).

The HE stained section was used to determine the effect of SNCP on the re-epithelialization and contraction of the wound surface at 7 days post-wounding (Figure 4a). The distance of double arrows and red arrows represent the width of the wound surface and the length of newly-formed epidermal, respectively. During the course of wound contraction and re-epithelialization in the early stage of wound healing, the width of wound surface of SNCP group (1.99 ± 0.07 mm) was significantly smaller than that of the NC group (4.58 ± 0.48 mm) and the PC group (3.51 ± 0.21 mm) (Figure 4b) and the lengths of the newly-formed epidermal of SNCP group and PC group were (3.00 ± 0.39 mm) and (2.47 ± 0.24 mm), respectively, which are significantly longer than that of the NC group (1.26 ± 0.42 mm) (Figure 4b). As shown in Figure 4c, compared with the NC group, the SNCP group and the PC group reduced the mRNA levels of TGF-β1, TNF-α and IL-1β, and the mRNA level of TNF-α in the SNCP group was significantly lower than that in the PC group (*p* < 0.01). The decrease of these pro-inflammatory factors indicated that SNCP possessed an anti-inflammatory function, which was beneficial to rapid wound healing and the prevention of abnormal scar formation.

### 2.7. SNCP Improves the Healing Quality of Wound Epidermis 

**Figure 5:**
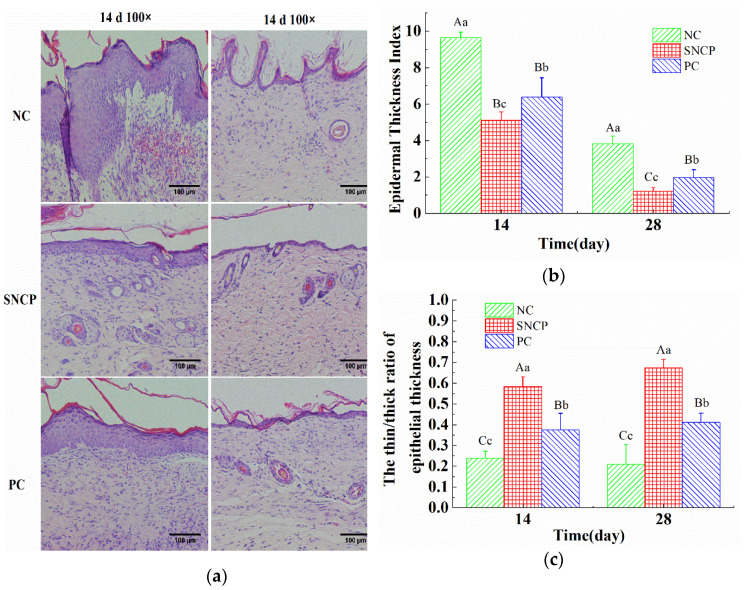
Effect of SNCP on the healing quality of wound epidermis at 14 days and 28 days post-wounding. (**a**) Histological analysis of the healing quality of wound epidermis, (scale bar = 100 μm). (**b**) Epidermal thickness index of wound epidermis, n = 4. (**c**) Thin/thick ratio of epithelial thickness of wound epidermis, n = 4. Values with different letters indicate significant differences in the different groups (capital letters: *p* < 0.01, small letters: *p* < 0.05).

A thinner and flatter epidermis was observed in the SNCP group and PC group at 14 and 28 days post-wounding, while an uneven and thickened epidermis with tongue-like protrusions on the edges was observed in the NC group (Figure 5a). The epidermal thickness index of the SNCP group and PC group were (1.23 ± 0.18) and (1.96 ± 0.44), which were significantly lower than in the NC group (3.82 ± 0.42) at 28 days post-wounding (Figure 5b). An epidermal thickness index of more than 1 means that the epidermis was thicker than normal skin. The thickness ratio of the thinner and thicker epidermis (thin/thick ratio) was used to characterize whether the epidermis was recovered uniformly and flatly. At 28 days post-wounding, the thin/thick ratio of the epidermis in the SNCP group and PC group were (0.67 ± 0.04) and (0.41 ± 0.04), respectively, which were significantly higher than in the NC group (0.21 ± 0.10) (Figure 5c).

### 2.8. SNCP Improves Collagen Deposition and Recombination at the Wound Dermis 

**Figure 6:**
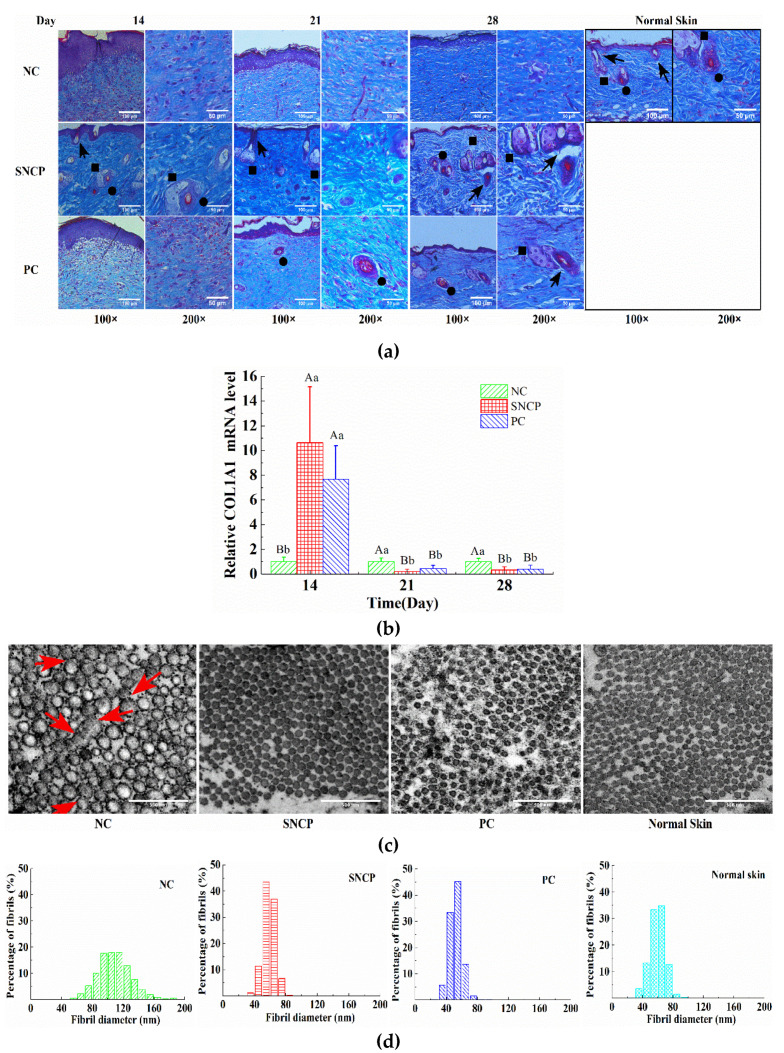
The effect of SNCP on collagen deposition and recombination at the wound dermis. (**a**) Masson-stained section analysis of collagen deposition and recombination at the wound dermis; the circle, arrow and square indicate a sebaceous gland, hair follicle and blood vessel, respectively. Images shown at ×100 magnification (scale bar = 100 μm) and ×200 magnification (scale bar = 50 μm). (**b**) Expression of type I collagen (COL1A1) in wound of mice skin, n ≥ 4. Values with different letters indicate significant differences in the different groups (capital letters: *p* < 0.01, small letters: *p* < 0.05). (**c**) Transmission electron microscopy (TEM) analysis of cross-sectional collagen fibers (at ×5000 magnification), scale bar = 500 nm; focal lateral fusion and unclear outline (red arrow) are indicated. (**d**) Diameter distribution of collagen fibers from wounds at 21 days post-wounding, n ≥ 1200.

As shown in Figure 6a, the formation of skin appendages (such as hair follicles, sebaceous glands and blood vessels) were not observed in the NC group at 14, 21 and 28 days, and the collagen was mainly arranged in parallel. However, blood vessel formation and collagen deposition were observed at the dermis of PC group at 21 days post-wounding, and a basket-like collagen distribution was formed at a later stage. Compared with the NC group and the PC group, sebaceous glands were produced on the dermis of the SNCP group at 14 days, and mature granulation tissue, including blood vessels, hair follicles and sebaceous glands, was produced at 21 days, which greatly improved collagen deposition and formed an orderly arrangement of a basket weave-like structure, and gradually showed the characteristics of normal dermis. The dermis of the SNCP group was similar to the normal healthy skin at 28 days (Figure 6a). In addition, the COL1A1 level in wound tissue of mice treated with SNCP was significantly higher than that of NC group on the 14th day after injury (*p* < 0.01), while the COL1A1 level was significantly down-regulated on the 21st and 28th day (*p* < 0.01) (Figure 6b).

To further observe the collagen fibers during tissue remodeling, the ultrastructure of collagen fibers in the wound was identified by transmission electron microscope (TEM) at 21 days post-wounding. The average diameter of collagen fibers in dermis of the NC group, the SNCP group, the PC group and normal skin were 108.7 nm, 58.9 nm, 52.3 nm and 59.5 nm, respectively. The SNCP and PC groups showed a similar appearance to normal skin, with relatively round collagen fibers and clear contours, while the NC group show obvious structural abnormalities (indicating by the red arrow in Figure 6c), including unclear contours between multiple collagens, tending to focal lateral fusion to form larger collagen fibers. As shown in Figure 6d, the fiber diameter distribution in skin of the SNCP, PC and normal skin groups were relatively concentrated, ranging from 30 mm to 80 nm, while the NC group had a wide distribution range (60–160 nm).

### 2.9. SNCP Blockades the TGF-β/Smad Signaling Pathway to Achieve Scarless Healing

**Figure 7:**
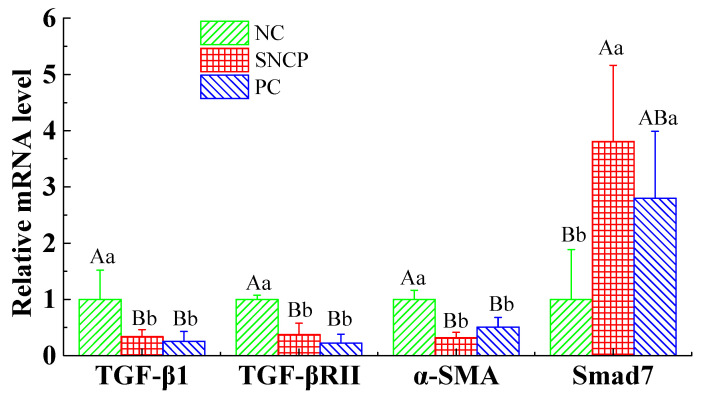
Expression of several factors related to TGF-β/Smads signaling pathway. Values with different letters indicate significant differences in the different groups (capital letters: *p* < 0.01, small letters: *p* < 0.05).

To investigate the effect of SNCP on TGF-β/Smad signaling pathway, the mRNA expression of transforming growth factor-β1 (TGF-β1), transforming growth factor-β receptor II (TGF-βRII), α-smooth muscle actin (α-SMA) and Smad7 in the wound at 28 days post-wounding, were investigated by qRT-PCR. As shown in Figure 7, compared with the NC group, the mRNA levels of Smad7 in both the SNCP group and PC group were up-regulated; in contrast, the mRNA levels of TGF-βRII, COL1A1 and α-SMA were down-regulated, with a statistically significant difference (*p* < 0.01), but there was no statistically significant difference between the SNCP group and PC group (*p* > 0.05).

## 3. Discussion

Our previous studies had found that the *Sipunculus nudus* extract had been shown to have obvious activity of accelerating wound healing [6]. *Sipuncula* is rich in collagen [11,26], and collagen hydrolysate is considered to be an effective active substance for wound repair. To investigate functions of collagen peptides in *Sipunculus nudus*, the physical and chemical properties of collagen (SNC) and the effects of collagen peptides (SNCP) on promoting wound healing were studied. The results show that SNC was a typical type I collagen with a denaturation temperature of 32.8 °C (Figure 1), while SNCP possessed an outstanding capacity to induce HUVEC, HaCaT and HSF cells proliferation and migration (Figure 2). Through the use of the mouse skin full cortical wound model, we demonstrated that the SNCP had a potential activity of accelerating wound healing and preventing pathological scar formation (Figure 3), and its mechanism was related to regulation of inflammatory factors, collagen deposition, collagen fiber remodeling and blockade of the TGF-β/Smads signal pathway. Our findings provide a theoretical basis for the wound healing potential of SNCP.

Nutrition is an important part of wound healing, and maintaining good nutrition could be one of the effective strategies to promote a more rapid wound healing [3]. Derived from collagen of *Sipunculus nudus*, SNCP was consisted of collagen peptides and free amino acids, providing a nutrient matrix for wound healing. Collagen peptides exert a variety of biological activities. In the skin, collagen peptides act as false collagen degradation peptides, which send a false signal in the fibroblast cells to synthesize new collagen fibers. Moreover, they possess chemotactic properties to promote cell migration and proliferation, which is an important process in wound healing [7,15,17]. In this study, SNCP was mixed collagen peptides with different molecular weights, containing a large number of low molecular weight peptides, of which components of less than 5 kDa accounted for approximately 81.95%. It has been proved that absorption of collagen peptide was improved by smaller size [3]. Therefore, the advantage of wound healing process by applying SNCP may also be due to its small peptides, which facilitate body absorption. Moreover, essential amino acids provide excellent nutritional support for wound healing [3]. L-arginine has been shown to enhance wound strength and collagen deposition in rodents and humans, as a supplementation accelerating the diabetic wound healing [19,39]. The SNCP is rich in Arg with 0.81 g/100 g (accounting for 19.36% of the total) (Table 1), which might contribute to the potential wound healing effect. However, the precise functional components of SNCP remained unclear. In the future, the separation and functional evaluation of collagen peptides with different MW size are needed to confirm the active components in SNCP. 

The wound healing process takes place in distinct phases that at some times may overlap, proceeding by a preliminary hemostatic phase, following by the inflammatory phase, the proliferating phase and the maturation one [3,4]. In the inflammatory period, inflammation is necessary for resisting the invading environmental pollutants and removing tissue debris. However, excessive inflammation could cause damage to the normal tissues, which was the main cause of disease related to fibrosis [40]. It had been reported that both fetal trauma and oral mucosal trauma showed relatively low inflammation, and both of them could quickly seal the wound without scars [41,42]. Many experimental models of wound repair showed that excessive inflammation could delay healing and cause scar formation [43]. IL-1β and TNF-α are important inflammatory cytokines and indicators of inflammatory response. In this study, SNCP could significantly decrease the level of IL-1β and TNF-α in the wound surface at 7 days post-wounding, which indicates SNCP processed activity of reducing inflammation (Figure 4c). In addition, IL-1β and TNF-α could reduce collagen synthesis in a dose-dependent manner, among which TNF-α strongly promoted matrix metalloproteinase (MMP) secretion, degraded collagen and blocked the mRNA transcription of type I/III pro-collagen [44]. This might also be a cause of lower mRNA levels of COL1A1 in the NC group at 14 days (Figure 6b). TGF-β1 was an important driver of fibrosis process, which was related to the formation of abnormal scars [45]. In fetal wounds, the addition of exogenous TGF-β1 resulted in the same scarring effect as adults [46,47]. At 7 days post-wounding, the level of TGF-β1 mRNA in SNCP group was lower than that in NC group, which indicate that SNCP might have a better anti-scar activity. HE-stained sections confirm these views (Figure 4a and Figure 5a). The epidermis of SNCP group was thin and flat, while the epidermis of NC group was fat, thickened and uneven, with a tongue-like edge [48]. 

In the proliferating phase, the structural and functional reconstruction of differentiated muscle fibroblasts in granulation perform repair processes. Scarless healing is largely dependent on the reconstruction of normal ECM. During this period, the mature collagen was remodeled to form an ordered basket weaving arrangement, with a structure and appearance similar to normal skin [49]. As shown in Figure 6a, mature granulation tissues, such as sebaceous glands, blood vessels and hair follicles, were observed in the dermal layer of the SNCP group. However, lack of normal healthy skin appendages was observed in the NC group and the collagen gathered in parallel to form a bundle, which was excessively accumulated and easily formed scars. In addition, the SNCP group could regulate the formation of collagen. The expression of COL1A1 mRNA was up-regulated at 14 days (Figure 6b), which indicates that SNCP could promote the synthesis of a large amount of collagen to form ECM during the proliferation period. However, during the remodeling period, COL1A1 mRNA maintains a relatively low level, which allowed the dermis to maintain a dynamic balance between collagen synthesis and decomposition. The tissue degraded excessive ECM including abnormal collagen while synthesizing new mature collagen to reshape ECM, preventing too much immature collagen from accumulating into large linear bundles to form scars or keloids [50]. In this study, the expression of COL1A1 mRNA in SNCP groups was down-regulation at 21 days post-wounding (Figure 6b). In addition, TEM analysis also confirmed that treatment with SNCP for 21 days show a similar appearance to normal skin, with relatively round and concentrated diameter distribution collagen fibers and clear contours (Figure 6c).

TGF-β/Smad signal transduction pathway is an important way to affect post-traumatic scar formation and extracellular matrix deposition [51]. In the TGF-β/Smad pathway, TGF-β1 acts as a growth factorial signaling molecule in wound healing and scar formation. When TGF-β1 binds to TGF-β type I and type II receptors (TβRI and TβRII, respectively) on the cell membrane to form the active signaling complex, the TGF-β mediated signal transduction is activated [51], which results in the persistent presence of high levels of α-SMA, causing excessive collagen deposition (COL1A1) and inducing an abnormal fibroblast phenotype, a unique sign of pathological scars [52]. Smad7, a negative feedback-regulated inhibitory protein, could inhibit Smad2/Smad3 phosphorylation and block the TGF-β signaling pathway by strongly binding to TGF-βRI to prevent pathological effects caused by TGF-β and inhibit scar hyperplasia [53,54,55]. In our study, compared with the NC group, the expression levels of TGF-β1 and TGF-βRII in the SNCP group were significantly down-regulated (*p* < 0.01), while the expression levels of Smad7 were significantly up-regulated (*p* < 0.01) (Figure 7), which inhibited and blocked the TGF-β/Smads signal transduction pathway. Those might be the reasons why SNCP could regulate collagen formation and remodeling, thereby preventing pathological effects caused by TGF-β and inhibiting scar hyperplasia.

## 4. Materials and Methods

### 4.1. Materials

Fresh *Sipunculus nudus* (diameter, 4–8 mm, length, 8–12 mm), was purchased from the Dongfeng Market of Zhanjiang, China. Coelomic fluid was removed, and the coelomic walls were obtained. Enzymatic hydrolysis of animal protease (2 × 10^5^ U/g) and flavor enzyme (2 × 10^5^ U/g) were purchased from Pangbo Biological Engineering Co., Ltd. (Nanning, China); Pepsin (1:10,000) was purchased from Yuanye Biotechnology Co., Ltd. (Shanghai, China).

### 4.2. Extraction of the Collagen and Preparation of Collagen Peptides 

To extract *Sipunculus nudus* collagen (SNC), all processes were conducted at 4 °C. The coelomic walls were fully cut and pasted, then soaked in 0.2 M NaOH for 24 h at a ratio of 1:10 (*w*/*v*) to remove non-collagenous proteins. The mixture was centrifuged at 10,000 *g* for 20 min. After removing the supernatant, the precipitation was washed repeatedly with distilled water, then soaked in 0.5 M acetic acid solution with 0.3% pepsin and homogenized at a ratio of 1:10 (*w*/*v*). After incubating for 48 h, the suspension was centrifuged at 10,000 *g* for 20 min (Heraeus Fresco Germany), and the supernatant was salted out by the addition of NaCl to a final concentration of 1 M. The mixture was allowed to stand overnight (at 4 °C), and the precipitates were collected (centrifugation, 12,000 *g*, 30 min), dissolved in 0.5 M acetic acid (10%), dialyzed (MD 34, USA) for 2 d and finally lyophilized (Labconco Freeze Dryer FreeZone 6 Liter, CA, USA). 

SNC was hydrolyzed by animal hydrolytic protease (3000 U/g) and flavor protease (3000 U/g) under the condition of pH 7.0, a solid/solution ratio of 1:300 (*w*/*v*) and at 55 °C for 5 h, and the mixture was heated to 100 °C for inactivation, followed by centrifugation. After lyophilization, *Sipunculus nudus* collagen peptide (SNCP) was obtained. 

### 4.3. Sodium Dodecyl Sulphate Polyacrylamide-Gel Electrophoresis (SDS-PAGE)

One μL sample loading buffer (5×) (Beyotime Institute of Biotechnology, Jiangsu, China) was dissolved in 4 μL the SNC sample. The mixture was heated to 100 °C for 5 min, followed by centrifuging at 9000 *g* for 5 min. Subsequently, 5 μL supernatant was loaded onto an instant polyacrylamide-gel made of 10% running gel and 6% stacking gel. After electrophoresis, the gels were stained BeyoBlue™ Coomassie Blue Super Fast Staining Solution and destained with deionized water. Quantitative analysis of protein band intensity was performed using a Model GS-700 Imaging Densitometer (Bio-Red Laboratories, Hercules, CA, USA) with Molecular Analyst Software version 1.4 (image analysis systems). 

### 4.4. Fourier Transform Infrared Spectroscopy (FTIR)

The chemical structure of SNC was studied by FTIR spectroscopy (Bruker Tensor 27 Instruments, Billerica, MA, Germany). The samples were prepared in agate mortar containing 3 mg collagen in approximately 150 mg potassium bromide (KBr). The spectra were obtained with wave numbers ranging from 4000 cm^−1^ to 400 cm^−1^ at a resolution of 2 cm^−1^ over a total of 32 scans. The background was subtracted by the Opus software (Bruker Instruments, Billerica, MA).

### 4.5. Differential Scanning Calorimetry (DSC)

The SNC (5 mg) was weighed accurately (0.1 mg) into aluminum pans and sealed. The samples were heated from 24 to 100 °C at a scanning rate of 1 K/min, with an empty sealed pan as a reference. The transition temperature (Tm) was recorded.

### 4.6. Determination of Molecular Weight Distribution of SNCP

The molecular weight distribution of the SNCP was determined by high performance liquid chromatography (Angilent 1200, Palo Alto, CA, USA) following the methods of other researchers [15]. The mixed standard samples, which consisted of cyyochrome (12,500 Da), aprotinin (6500 Da), bacitacin (1450 Da), ethyl amino acid-ethyl amino acid-tyrosine-arginine (451 Da) and ethyl amino acid-ethyl amino acid-ethyl amino acid (189 Da), were loaded into the column. Acetonitrile/water (45:55) with 0.1% trifluoroacetic acid was adopted as mobile phase, the flow rate was 0.5 mL/min and the UV wavelength was 220 nm. A standard curve of retention time absorbance was plotted. The SNCP solution was filtered through a 0.45 μm filter and subjected in the same conditions. Then, the molecular weight was calculated according to the retention time using the curve equation.

### 4.7. Amino Acid Composition Measurement of SNCP 

The amino acid of the SNCP was determined according to the previous study [56]. SNCP was hydrolyzed by dissolving it in 6 M HCl at 110 °C for 24 h. The filtrate and standard samples of amino acids solution was analyzed with an automatic amino acid analyzer (S-433D, Sykam, Bremen, Germany).

### 4.8. In Vitro Scratch Assay

The wound healing assay in vitro was performed by two-well wound-generating insert (Ibidi). After adding the wound-generating insert into a 24-well plate, 50 μL cell suspensions (5 × 10^5^ cells/mL HaCaT, 5 × 10^5^ cells/mL HSF, 1 × 10^5^ cells/mL HUVEC) were seeded per well of the insert with Dulbecco’s modified Eagle’s medium (DMEM; Hyclone), containing 10% (*v*/*v*) fetal bovine serum (FBS; Hyclone), 1% (*v*/*v*) penicillin and streptomycin (Hyclone), respectively. After incubation overnight to form a monolayer, the inserts were removed, after which the debris was washed with PBS twice. The monolayer cells were incubated in complete medium containing 10% PBS (*v*/*v*) as the negative control (NC) and SNCP with a concentration of 500 μg/mL. The image was observed and analyzed by using an inverted fluorescence microscope (DMI4 000, Leica, Germany) and the wound area was calculated with the Image J software (National Institute of Heath, Bethesda, USA). The wound closure percentage was obtained by the following formula:

Wound closure (%) = (A_0_ − A_t_)/A_0_ × 100, (1)

where A_0_ was the wound area at 0 h and A_t_ was the remaining area at the designated time.

### 4.9. In Vivo Wound Healing Assay

All animal programs in this study were approved by the Animal Ethics Committee of Guangdong Ocean University, and were conducted in accordance with the national regulations (the approval number assigned by the ethical committee for our animal experiment was GDOU-LAE-2019-0003). Kunming (KM) male mice aged 7 to 8 weeks were purchased from Sibefu Biotechnology Co., Ltd. (Beijing, China). The mice were anesthetized, and the area on the back was shaved and cleaned with ethanol before the surgery. Full-thickness excisional round holes with diameter of 8 mm were made on the exposed skin of the back using disposable biopsy punch (Rapid-Core, EMS, America). The group of mice with none-treatment wound was considered as negative control group (NC). Each 2 g SNCP (based on total contents of nitrogen) was added to 1 mL of sterile water to form an ointment for application to the wound in the SNCP group (SNCP), and the Yunnanbaiyao powder was applied to the wound in the positive control group (PC). In both cases were applied daily. Mice were placed in a single cage at a temperature of 25 °C and given enough food and water. 

### 4.10. Macroscopic Evaluation of Wounded Tissues

For measurement of wound closure percentage, wounds were photographed every 2 days post-wounding, and the wound closure percentage was obtained by the following formula:

Wound closure (%) = (S_0_ − S_t_)/S_0_ × 100, (2)

where S_0_ was the wound area at 0 day and S_t_ was the remaining area at the designated time.

### 4.11. Microscopic Evaluation of HE-Stained Sections

For histologic analysis, the surrounding skins of the wound were fixed in 4% formalin, embedded into paraffin, for the hematoxylin and eosin- (H&E; Bogu Biotechnology Co., Ltd. Shanghai, China) stained sections at day 7 post-wounding to measure the width of the wound surface and the length of the epithelial growth, for the H&E-stained sections at day 14 and 28 post-wounding to analysis the epithelial thickness index (ETI) and the thin/thick ratio of epithelial thickness and for the Masson’s trichrome-stained sections at day 14, 21 and 28 post-wounding to observe the synthesis and deposition of collagen, angiogenesis and the regeneration of hair follicle and sweat gland in dermal. The ETI and the thin/thick ratio of epithelial thickness were obtained by the following formula:

Epithelial thickness index (ETI) = δ_1_/δ_2_, (3)

Thin/thick ratio of epithelial thickness = δ_3_/δ_4_, (4)

where δ_1_, δ_2_, δ_3_ and δ_4_ refer to the average new-forming epidermal height, the average normal epidermal height, the new-forming thinner epithelial thickness and the new-forming thicker epithelial thickness, respectively.

### 4.12. Quantitative Real-Time Polymerase Chain Reaction (qRT-PCR) Analysis

Total RNAs were extracted from the skins of wound using RNAIso Plus (Takara). The cDNAs were synthesized using Prime ScriptTM RT reagent Kit with gDNA Eraser (Takara) in accordance with the manufacturer’s instructions. The qRT-PCR reaction conditions were 95 °C for 30 s, followed by 40 cycles of 95 °C for 5 s and 60 °C for 30 s. Expression levels of target genes were calculated using the comparative CT method with glyceraldehyde-3-phosphate-dehydrogenase (GAPDH) as an endogenous reference gene. To confirm amplification of specific transcripts, melting curve profiles were produced at the end of each reaction. The following primer sets were used (Table 2). 

### 4.13. TEM Analysis

The wounded tissues were fixed at 4 °C in 4% paraformaldehyde for 30 min, then in 2.5% glutaraldehyde for overnight, and subsequently in 1% osmium tetroxide for 2–3 h. Then, after washing, dehydrating, embedding and solidifying, transmission electron microscopy (TEM, Tecnai G2 20 TWIN; FEI) analyses were operated at an accelerating voltage of 200 kV and a magnification of 5000. Image J software (National Institute of Heath) was employed to analyze the diameter of each fibril in each image. 

### 4.14. Statistics Analysis

All experimental values were presented as the means ± the standard deviations (SD). The comparison analysis between the groups was assessed via one-way analysis of variance (ANOVA) with the The John’s Macintosh Product (Version 10, SAS, Cary, NC, USA). Statistical significance among groups was designated as follows: statistical significances of *p* < 0.01 and *p* < 0.05 between groups were presented via different capital letters and lowercase letters, respectively, while *p* > 0.05 was presented via the same lowercase letters among groups.

## 5. Conclusions

In this study, we found that SNCP could accelerate the wound healing and inhibit scar formation in a skin wound of mice, primarily by facilitating rapid proliferation of epithelial cells, reducing inflammation, improving collagen deposition and recombination and blockade of the TGF-β/Smads signal pathway. Therefore, SNCP may have promising clinical applications in skin wound repair and scar inhibition. However, further molecular mechanisms remain to be elucidated.

## Figures and Tables

**Table 1:** Amino acid composition of SNCP.

Amino Acids.	Free Amino Acid (g/100 g)	Free Amino Acid Residues per 1000 Residues	Hydrolyzed Amino Acids (g/100 g)	Hydrolyzed Amino Acid Residues per 1000 Residues
**Aspartic acid (Asp)**	0.04	10	4.09 *	40 *
**Threonine (Thr)**	0.19	52	4.75	51
**Serine (Ser)**	0.09	28	4.01	49
**Proline (Pro)**	0.34	96	4.00	45
**Glutamic acid (Glu)**	0.19	42	16.64 ^#^	145 ^#^
**Glycine (Gly)**	0.12	52	20.86	357
**Alanine (Ala)**	0.24	88	8.50	123
**Cysteine (Cys)**	0.03	8	0.58	6
**Valine (Val)**	0.18	50	1.58	17
**Methionine (Met)**	0.06	13	0.49	4
**Isoleucine (Ile)**	0.26	65	1.40	14
**Leucine (Leu)**	0.51	127	2.32	23
**Tyrosine (Tyr)**	0.42	76	0.08	1
**Phenylalanine (Phe)**	0.38	75	1.03	8
**Lysine (Lys)**	0.22	49	1.74	15
**Histidine (His)**	0.08	17	0.62	5
**Arginine (Arg)**	0.81	152	13.11	97
**Total**	4.16	1000	85.81	1000

* Asx (Asp + Asn); ^#^ Glx (Glu + Gln).

**Table 2:** Primers used for the qRT-PCR analysis.

Gene	Forward	Reverse
GADPH	CAGGAGGCATTGCTGATGAT	GAAGGCTGGGGCTCATTT
TNF-α	GGTCAATCTGCCCAAGTA	CACCCATTCCCTTCACAG
IL-1β	TATGGGCTGGACTGTTTCTAATGC	TTCTTGTGACCCTGAGCGACCT
TGF-β1	CCGCAACAACGCCATCTAT	CCAAGGTAACGCCAGGAAT
COL1A1	ACGCCATCAAGGTCTACTGC	GAATCCATCGGTCATGCTCT
α-SMA	AGACATCAGGGAGTAATGGTTG	GAAGCTCGTTATAGAAAGAGTGG
TGF-βRII	TGAGAAGCCGCATGAAGT	AGAGTGAAGCCGTGGTAGGT
Smad7	TTTACAACCGCAGCAGTTAC	GGCTGTAGGCTTTCTCATAGT

## Data Availability

All data presented in this study are available in the article.
